# A new concept for the genesis of felsic magma: the separation of slab-derived supercritical liquid

**DOI:** 10.1038/s41598-020-65641-6

**Published:** 2020-05-26

**Authors:** Hajime Taniuchi, Takeshi Kuritani, Tetsuya Yokoyama, Eizo Nakamura, Mitsuhiro Nakagawa

**Affiliations:** 1 Department of Natural History Science, Graduate School of Science, Hokkaido University, Sapporo, Hokkaido 060-0810 Japan; 2 Department of Earth and Planetary Science, Faculty of Science, Hokkaido University, Sapporo, Hokkaido 060-0810 Japan; 3 The Pheasant Memorial Laboratory, Institute for Planetary Materials, Okayama University, Misasa, Tottori 682-0193 Japan; 4 Department of Earth and Planetary Sciences, Tokyo Institute of Technology, Ookayama, Meguro, Tokyo 152-8551 Japan

**Keywords:** Geochemistry, Petrology, Volcanology

## Abstract

Felsic magmas produced at subduction zones have played an important role in the generation and evolution of the continental crust. For the origin of felsic magmas, processes such as fractional crystallization of mafic magmas, partial melting of crustal materials, partial melting of subducting slabs, and partial melting of pyroxenitic mantle wedge components have been proposed. Recent experimental studies have predicted that felsic melt can also be produced in the mantle wedge by the separation of slab-derived supercritical liquid beyond depths corresponding to the critical point. To date, however, the presence of felsic magma of this origin has not yet been reported. In this study, we investigated dacitic lavas and preceding calc-alkaline andesite lavas from the Rishiri Volcano, located at the rear of the Kuril arc. We show that hydrous felsic melt and aqueous fluid were separated from slab-derived supercritical liquid in the mantle wedge. The former erupted as dacitic magma whilst the aqueous fluid induced the generation of primary basaltic magma involved in creating calc-alkaline andesite magma. We infer that slab-derived supercritical liquid is an efficient transport medium for moving silicate-rich components from subducting slabs to the Earth’s surface, and that this process may have contributed to the growth of the continental crust.

## Introduction

Subduction-zone magmatism is considered to have played an important role in the generation and evolution of the continental crust, because the chemical composition of calc-alkaline andesites that are widespread in island arcs and active continental margins is similar to the average composition of the continental crust^1,2^. Primary mafic magmas are thought to be generated by melting of the mantle wedge with an influx of slab-derived water-rich materials^3,4^. Intermediate to felsic magmas (andesite to rhyolite) are also commonly produced in many subduction zones. Some felsic magmas have been emplaced as large granitic plutons, which contribute to the establishment of a buoyant continental crust^5,6^, and others have mixed with mafic magmas to produce calc-alkaline andesitic magmas and other intermediate magmas in crustal magma chambers^7^, producing geochemical variability in the crust^2^.

It has been suggested that felsic magmas found at subduction-zone volcanoes are produced by mechanisms including fractional crystallization with or without crustal assimilation^8^, partial melting of the crust^9,10^, and partial melting of metasomatised silica-excess pyroxenite mantle^11,12^. In addition, adakitic magmas with high-Sr/Y ratios^13^ and high [La/Yb]_N_ (primitive mantle-normalised La/Yb) ratios^14,15^ can also be produced by partial melting of hot subducting slabs at young subduction zones^13–15^ and by partial melting of garnet-bearing lower part of the thick crust^16,17^.

Recently, experimental studies have suggested that intermediate to felsic magma may also be produced as hydrous melt through the separation of slab-derived supercritical liquids in the sub-arc mantle. At relatively shallow depths, the slab-derived materials are released as aqueous fluid or hydrous melt. However, with increasing depths of fluid release, the solubility of silicate materials in aqueous fluid tends to become close to those of water in silicate melt, and the slab-derived water-rich materials would eventually become supercritical liquids at the critical point. On the other hand, ascending slab-derived supercritical liquid would separate into aqueous fluid and hydrous melt at the critical point^18,19^. Because the pressure corresponding to the critical end-point for silicic supercritical liquid can be as low as <3 GPa, andesitic to dacitic magmas would be generated by the separation of slab-derived supercritical liquid in sub-arc mantle^20^. To date, however, the presence of magmas originating from this process has not yet been reported in nature.

**Figure 1:**
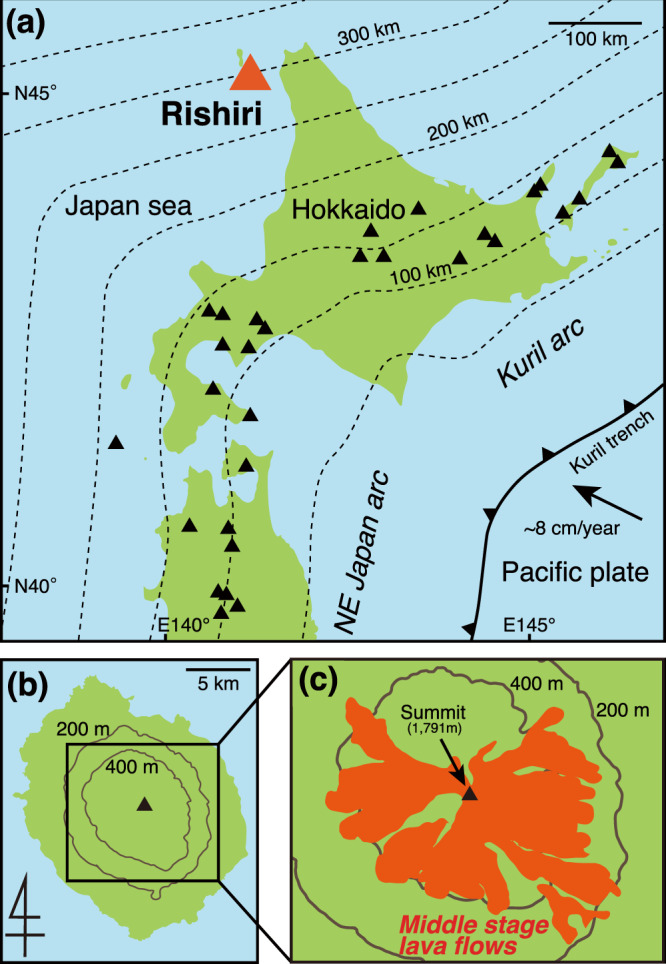
(**a**) The location of the Rishiri Volcano, (**b**) map of Rishiri island, and (**c**) the distribution of Middle Stage^25^ lava flows, including the studied dacite and andesite. In (**a**), the thin grey dashed lines denote slab surface contours, and the solid triangles indicate locations of active volcanoes. The slab surface contours, and the subduction direction are from ref. ^52^. In (**c**), distributions are from ref. ^25^.

Rishiri Volcano is a Quaternary stratovolcano at the rear of the southern Kuril arc, located 300 km above the Wadati-Benioff Zone (Fig. 1a). Previous studies have suggested that slab-derived supercritical liquid has been released from the subducting slab^21,22^, and that some dacitic lavas have adakitic geochemical signatures^23^. In this study, we conducted a detailed petrological, geochemical, and chronological study on the dacites. We suggest that the dacitic magmas represent hydrous melts generated through separation of slab-derived supercritical liquid.

## Results

**Figure 2:**
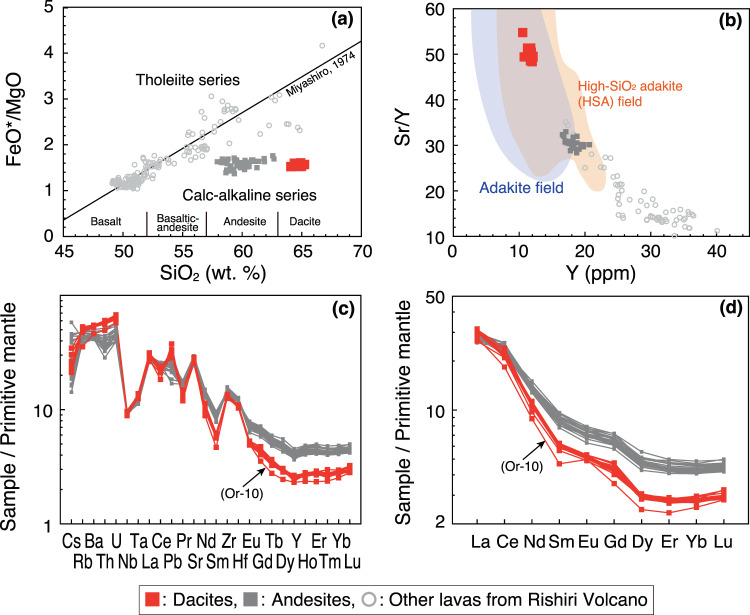
Composition of the dacite lavas from the Rishiri Volcano, shown in (**a**) FeO*/MgO–SiO_2_ diagram, (**b**) Sr/Y–Y diagrams, (**c**) primitive mantle-normalised diagram of trace element concentration, and (**d**) primitive mantle-normalised rare-earth element concentration. In (**a**), the discrimination line between the calc-alkaline and tholeiite series is taken from ref. ^26^. In (**b**), the compositional fields of the High-SiO_2_ adakite (HSA) and adakite are taken from ref. ^15^ and ref. ^13^, respectively. In (**a–d**), the data of refs. ^23,53^ are also shown. In (**c,d**), the trace element concentrations of primitive mantle are from ref. ^54^.

The Rishiri Volcano is characterised by the coexistence of alkali basalt, tholeiitic andesite to dacite, and calc-alkaline andesite to dacite^24^ (Fig. 2a and Supplementary Fig. S1a). Its volcanic activity can be divided into five stages: the Early-1, Early-2, Middle, Late-1, and Late-2 Stages^25^. The eruption rates of the individual stages are estimated to be >0.1 km^3^/ky, >0.4 km^3^/ky, >0.4 km^3^/ky, >0.35 km^3^/ky, and >0.09 km^3^/ky, respectively^25^. The main target of this study are the calc-alkaline dacitic lavas, which constitute the main edifice of the volcano together with the calc-alkaline andesite lavas and pyroclastics^25^ (Fig. 1b,c). These dacites and andesites belong to the Middle Stage of volcanic activity, characterised by the highest eruption rate over the lifetime of the volcano^25^. The petrogenesis of the calc-alkaline andesites was investigated in detail by ref. ^23^. The magmas were suggested to have been produced via mixing between crust-derived felsic magmas and primitive basaltic magmas generated through fluid-fluxed melting of the mantle. The dacitic lavas always overly the andesitic lavas in the area (Supplementary Fig. S2), indicating that the eruption of the andesitic magma predated that of the dacitic magma. New ^40^Ar/^39^Ar dating shows that activity leading to production of the dacite and andesite took place at 35.5 ± 1.4 and 34.6 ± 3.0 ka, respectively (Table S1 and Supplementary Fig. S3).

**Figure 3:**
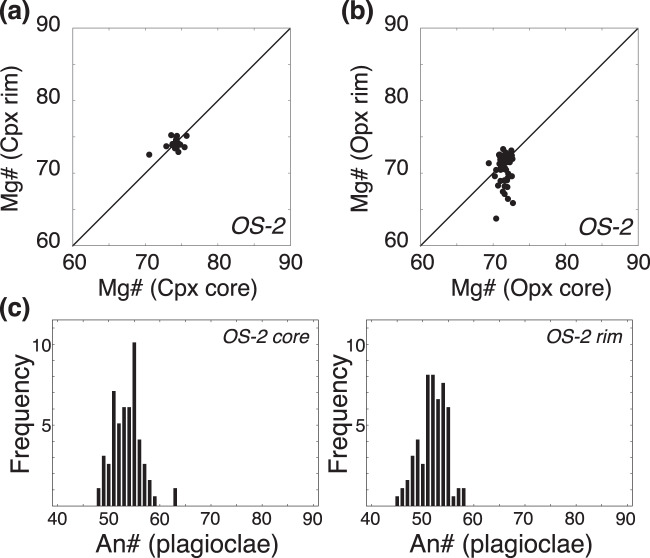
Compositions of (**a**) clinopyroxene and (**b**) orthopyroxene phenocrysts and (**c**) histograms of the An# contents of the cores and rims of plagioclase phenocrysts in the dacitic samples.

**Figure 4:**
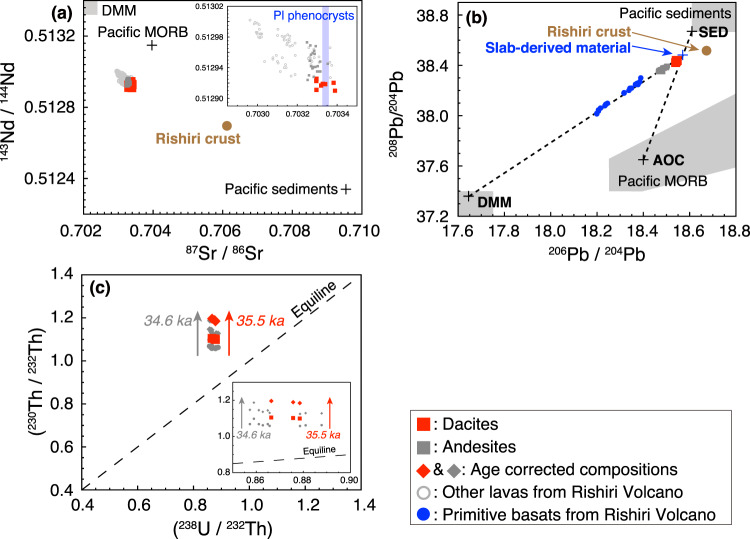
Composition of the dacite lavas from the Rishiri Volcano, shown in (**a**) the ^143^Nd/^144^Nd–^87^Sr/^86^Sr diagram, (**b**) the ^208^Pb/^204^Pb–^206^Pb/^204^Pb diagram, and (**c**) the U-Th equiline diagram. In (**a**,**b**), blown filled circles show the composition of crustal xenolith from the Rishiri Volcano^31^. In (**a**), the ^87^Sr/^86^Sr ratios of the plagioclase phenocrysts are also shown. In (**a,b**), the compositional fields of the depleted MORB mantle (DMM) are taken from ref. ^55^, and those of the Pacific sediments and Pacific MORB are from ref. ^56^. The representative compositions of the Pacific sediments (SED), Pacific MORB (AOC), and DMM are taken from refs. ^57,58^. In (**a,b**), the data of refs. ^21–23,31,53,59^ are also shown.

The phenocryst assemblage of the dacite lavas is composed of clinopyroxene, orthopyroxene, and plagioclase, some of which form crystal aggregates (Supplementary Fig. S4). All phenocrysts are clear under a polarised light microscope, and sieve-textured plagioclase is not found. The phenocrysts are homogeneous or normally zoned, with limited variation in their core composition (Fig. 3). The whole-rock SiO_2_ content of the dacites ranges from 64.1 to 65.3 wt.% (Table S2, Fig. 2a and Supplementary Fig. S1). The dacites and the preceding andesites belong to the calc-alkaline series in the Miyashiro diagram^26^ (Fig. 2a). The dacitic lavas have a relatively high MgO (2.3–2.7 wt.%; Supplementary Fig. S1e), Cr (25–42 ppm; Supplementary Fig. S1f), and Ni (26–39 ppm) content compared with other felsic lavas from the Rishiri Volcano. The Mg#’s [= Mg/(Mg + Fe^total^) × 100] of the dacites of 52–54 are similar to the average continental crust (~55; ref. ^2^), and are lower than those of mantle-derived primary melt (>60). The dacitic lavas are also characterised by high Sr/Y (48–55) and [La/Yb]_N_ (9–11) ratios, and low Yb_N_ (2.5–3.0) (i.e., adakitic signature^13,14^) compared with the andesitic lavas (Fig. 2b–d), and have been classified as high-SiO_2_ adakite^15^ (Fig. 2b and Supplementary Fig. S1e). No significant Eu anomaly is observed in the dacite lavas (Fig. 2d), with the exception of one outlier sample with extremely low P_2_O_5_, Y, and Yb content (Supplementary Fig. S5). The dacitic lavas have higher ^87^Sr/^86^Sr, ^208^Pb/^204^Pb and ^206^Pb/^204^Pb ratios and lower ^143^Nd/^144^Nd ratios than both the calc-alkaline andesitic and basaltic lavas from the volcano (Fig. 4a,b). The Pb isotopic compositions of the dacitic lavas are mostly on the binary mixing line between sediments (SED) and altered oceanic crust (AOC) (Fig. 4b). In a U–Th equiline diagram, age corrected compositions of the dacitic and andesitic lavas (Table S3) plot on the left-hand side of the equiline (Fig. 4c).

## Discussion

All phenocrysts in the dacite lavas are clear under a polarised light microscope, and essentially homogeneous in composition or slightly normally zoned with unimodal distributions in their core composition (Fig. 3). In addition, sieve-textured plagioclase is not found. These observations suggest that the dacitic magma did not undergo mixing after phenocryst crystallisation. The phenocryst phases occasionally form crystal aggregates, showing evidence of simultaneous growth, while no Eu anomaly is evident in whole-rock samples (Fig. 2d). In addition, the ^87^Sr/^86^Sr ratios of the plagioclase phenocrysts (Table S4) are identical to those of the host lavas (Fig. 4a). This indicates that all the phenocrysts were grown *in situ* in the dacitic magmas with no Eu anomaly.

The dacitic lavas are characterised by high Sr/Y and [La/Yb]_N_ ratios (Fig. 2b–d). One possible origin of dacitic melt with an adakitic signature is crystal fractionation, specifically of hornblende and garnet, from hydrous basaltic magmas^27^ or from calc-alkaline andesite magmas (with relatively lower [La/Yb]_N_ ratios; Fig. 2d) that erupted just before the dacite magmas. If this was the case, the dacitic melt would have been in equilibrium with hornblende or garnet at depth. The water content and temperature of the dacitic melt are estimated to be 5.3 ± 1.1 wt.% and 980 °C, respectively, at 0.46 GPa, using plagioclase-melt equilibrium (see Methods). Experimental studies^27^ showed that, for a dacitic melt with major element composition similar to that of the Rishiri dacite, the melt with ~5.3 wt.% H_2_O cannot be in equilibrium with hornblende unless the temperatures are lower than 930 °C at 0.4 GPa and 900 °C at 0.96 GPa. The melt cannot crystallize garnet at any temperatures at ≤0.96 GPa. Therefore, it is not likely that hornblende and/or garnet crystallised as a primary liquidus phase from the dacitic melt with ~980 °C in the crust (<~0.8 GPa; ref. ^28^) beneath Rishiri. This is supported by the absence of hornblende and garnet phenocrysts in the dacitic lavas, as well as in the calc-alkaline andesite lavas. Therefore, we conclude that the dacitic magmas were not derived from hydrous basaltic magmas or calc-alkaline andesite magmas through crystal fractionation.

It has also been recognised that felsic magmas with an adakitic signature can be produced by the partial melting of the lower part of the thick (>30 km) crust, consisting of garnet-bearing mafic rocks^16,17^. However, the adakitic signature could not be produced in the crust beneath the Rishiri Volcano, because the crust is too thin (~25 km; ref. ^28^) for the garnet to be stable^29,30^. The isotopic composition of dacitic lavas are found to be significantly different from the crustal xenoliths at the volcano^31^ (Fig. 4a,b), also negating the potential for the dacite magma to have originated from partial melting of the crust.

Based on these considerations, we conclude that the dacitic melt with adakitic signature could not have been derived from crustal magmatic processes but instead originated in the upper mantle. As it is unlikely that primary melt with dacitic composition was produced by the partial melting of mantle peridotite, the possible origin of the dacitic melt would be (1) partial melting of the subducting slab^13–15^, (2) partial melting of a pyroxenite mantle source with an influx of slab-derived fluid^11,12^, or (3) separation of slab-derived supercritical liquid^18–20^. These hypotheses are evaluated below.

If scenario (1) was the case, extremely high temperature would have been required to melt the slab, because the depth of the slab is ~300 km beneath Rishiri. Experimental studies have suggested that extremely high temperatures of >1300 °C are required to induce hydrous partial melting of the slab at the depth of 300 km^32,33^. However, the slab surface temperature are estimated to be ~1000 °C at 300 km depth, based on the extrapolation of the subduction thermal model (D80)^34^. In addition, evidence for temperature elevation in the slab, such as a slab window or slab tear, has not been found beneath the Rishiri Volcano^35,36^, suggesting that this scenario (1) is unlikely.

In scenario (2), the generation of dacitic melt is explained by partial melting of silica-excess pyroxenites, formed by the reaction of a peridotite mantle with infiltrated silicic slab components, in the sub-arc mantle^11,12^. In this case, it is expected that the primary dacitic melt would have high Ni contents^11,12^. However, the Rishiri dacites have significantly lower Ni contents of 26–39 ppm than those of mantle-derived primary magma (>~200 ppm). High-Ni olivines, that characteristically occur in magmas generated by the melting of a pyroxenite mantle source^11,12^, are also not observed in the dacite lavas. The Pb isotopic ratios of the dacite lavas can be explained by binary mixing between AOC component and SED component with little contribution of the depleted-MORB mantle (DMM) component (Fig. 4b); this observation also suggests that the mantle component was not significantly involved in the genesis of the dacites. For these reasons, we conclude that scenario (2) cannot explain the genesis of the dacitic magmas at Rishiri.

At the depth of the subducting slab of ~300 km at the Rishiri Volcano, supercritical liquid rather than aqueous fluid is expected to be released from the slab^20,32,33^. In fact, some primitive alkali basalt magmas at Rishiri are considered to have been generated by the melting of the source mantle with an influx of slab-derived supercritical liquid^21,22^. These alkali basalt lavas show ^230^Th-excesses with respect to ^238^U^21^. This is contrary to the U-excess signatures of frontal-arc lavas, which reflect a much higher mobility of U in slab-derived aqueous fluids than Th. Slab-derived supercritical liquids produce a ^230^Th-excess^21^ because of a preferential partition of Th relative to U^37,38^ during the dehydration of the subducting slab. The dacite lavas, as well as the preceding calc-alkaline andesitic lavas, show Th-excesses (Fig. 4c), which is consistent with the inference that slab-derived supercritical liquid was involved in the generation of both the dacite and andesite magmas. The eruption ages of the andesitic and dacitic lavas of 34.6 ± 3.0 and 35.5 ± 1.4 ka, respectively, and the stratigraphic relationship between the two indicate that the andesitic magmas erupted just before the dacitic magmas. In addition, the primitive basaltic magmas involved in the generation of the calc-alkaline andesitic magmas are considered to have been generated by the influx of slab-derived fluids into the source mantle^23^. These observations suggest that, in the upper mantle directly under the volcano, slab-derived fluid was present alongside the dacitic melt. As aqueous fluid and hydrous silicate melt cannot be produced from a subducting slab at an identical temperature^32,33^, these two components were most plausibly generated by the separation of ascending slab-derived supercritical liquid in the subarc mantle (i.e., scenario 3). The similarity of ^238^U–^230^Th disequilibria in the andesite and dacite lavas is also consistent with the derivation of andesites and dacites by essentially the same slab-derived supercritical liquid. The adakitic signature of the dacites (i.e., the silicate melt component in the slab-derived supercritical liquid) is considered to reflect the presence of residual garnet in the slab, because the basaltic oceanic crust is in the eclogite-facies field at a depth of ~300 km^39^.

**Figure 5:**
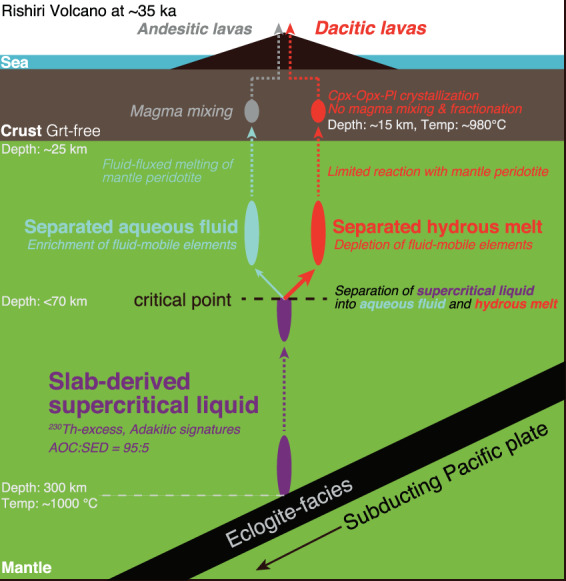
Schematic of the dacite magma generation at the Rishiri Volcano. See text for details.

From these considerations, this study concludes that the dacitic melt was most plausibly produced by the separation of slab-derived supercritical liquid^18–20^, rather than by the partial melting of the subducting slab^13–15^ or the partial melting of the pyroxenite mantle^11,12^. Slab-derived supercritical liquid ascending in the mantle wedge separated to produce hydrous melt and aqueous fluid beyond depths corresponding to the critical point. The former is represented by the dacitic melt and the latter induced generation of the primary basaltic magma involved in the generation of the calc-alkaline andesite magma (Fig. 5). It is difficult to estimate the depth at which the superciritical liquid was separated into the aqueous fluid and hydrous felsic melt, because of the scarcity of experimental studies. However, the high-K/Na alkali basalt magmas (Nozuka lavas) at Rishiri are considered to have been generated at ~2.3 GPa through an influx of slab-derived supercritical liquid into the melting region^22^. This observation suggests that the depth of the critical point for the supercritical liquid would have been shallower than the depth corresponding to ~2.3 GPa. If so, the separation of the slab-derived supercritical liquid for the dacite magmas might have occurred at depths corresponding to <~2.3 GPa.

The concentrations of fluid-mobile elements, such as Rb, Sr, Ba, and Pb, are significantly depleted in the dacitic lavas compared with the estimated concentrations of possible slab-derived supercritical liquid (see Supplementary Methods and Fig. S6). For example, the Rb/Zr and Pb/Hf ratios of the dacitic lavas of ~0.2 and ~1.6, respectively, are much lower than those of the supercritical liquid at 1.5 and 5.3, respectively. These features are consistent with preferential partitioning of fluid-mobile elements into an aqueous fluid relative to the remaining hydrous silicate melt during the separation of supercritical liquid^40^, although these features may also be attributed to a low content of fluid-mobile elements in the slab-derived supercritical liquid, which would have resulted from depletion in the subducting slab by removal through dehydration processes before reaching ~300 km depth.

Based on experimental studies on the behavior of slab-derived supercritical liquid^18,19^, Kawamoto *et al.*^20^ suggested the generation of two different types of primary mafic magmas in the mantle wedge beyond depths corresponding to the critical points; mafic magma from melting of the mantle with an influx of separated aqueous fluid and mafic magma from a reaction of separated hydrous melt with mantle peridotite. The occurrence of mafic magmas of these origins was suggested at the Mariana arc^41^. As such, this study suggests for the first time that hydrous felsic melt generated through the separation of slab-derived supercritical liquid can erupt without significant interaction with the mantle wedge.

Felsic magmas with high Sr/Y and [La/Yb]_N_ ratios have occurred as adakites in modern hot subduction zones^13^. It has been experimentally suggested that the minimum depth at which a supercritical liquid can occur in subducting slabs tends to become shallower with increasing proportions of sediment components in the slab^20^. The proportion of sediment components in adakites, such as those from the Southwestern Japan arc above the Philippine Sea slab, are ~40%^42^, significantly higher than studied dacites of ~5% (Supplementary Methods) above the Pacific slab. Moreover, the Philippine Sea slab is significantly hotter than the Pacific slab^43^. Thus, the supercritical liquid may have occurred at much shallower levels in the hot modern subducting slabs such as the Philippine Sea slab than the Pacific slab beneath the Rishiri Volcano. If so, some modern adakitic magmas may represent felsic magmas generated through the separation of slab-derived supercritical liquid.

Supercritical liquid may ascend in the mantle wedge more effectively than silicate melt, because of the reduced viscosity. The large ^230^Th-excess (Fig. 4c) of dacites from the Rishiri Volcano is consistent with the rapid transport of slab materials as supercritical liquid, although the timescale of transport cannot be estimated due to the uncertainty of the U/Th ratio of the slab source materials. We propose that slab-derived supercritical liquid is an efficient transport medium for silicate-rich components from the subducting slab through the mantle wedge to the surface, and it may have contributed to the growth of the continental crust.

## Methods

### ^40^Ar/^39^Ar age analyses

For reliable ^40^Ar/^39^Ar dating, we collected samples with holocrystalline groundmass. The ^40^Ar/^39^Ar age for groundmass of dacite and preceding andesite are obtained at the Argon Geochronology Laboratory, Oregon State University via incremental heating of hand-picked holocrystalline groundmass samples with laser irradiation. The ^40^Ar/^39^Ar isotopes are analysed by a multi-collector mass spectrometer (Thermo Scientific Model ARGUS-VI) and decay constants in this study from ref. ^44^ with 2σ internal errors.

### Whole-rock compositional analyses

Concentrations of whole-rock major elements and some trace elements (Sc, V, Cr, Co, and Ni) are determined via X-ray fluorescence (XRF) using a Spectoris MagiX PRO at the Graduate School of Science, Hokkaido University, and a Phillips PW2400 at the Pheasant Memorial Laboratory, Institute for Planetary Materials, Okayama University at Misasa^45^. Additional trace elements are analysed through inductively coupled plasma mass spectrometry (ICP-MS), using a Thermo Fisher Scientific X-series instrument at Hokkaido University and a quadrupole-type Yokogawa Agilent 7500cs system at Okayama University. The isotopic ratios for Sr, Nd, and Pb are determined using a multiple collector (MC)-ICP-MS (Neptune plus, Thermo Fisher Scientific) at Hokkaido University and a thermal ionisation mass spectrometer (TIMS) (Finnigan MAT 262) at Okayama University. Isotope analyses of U and Th are conducted by a TIMS (Finnigan MAT262 with RPQplus) at Okayama University. In this paper, isotopic ratios in parentheses represent activity ratios. Decay constants of the U and Th nuclides^46–48^ used for calculations in this study are λ_238U_ = 1.55125 × 10^−10^, λ_234U_ = 2.8263 × 10^−6^, λ_232Th_ = 4.9475 × 10^−11^, and λ_230Th_ = 9.158 × 10^−6^. The details of the analytical procedures at Okayama University, including those of the chemical separations and data correction, reflect those presented by ref. ^21^, and those at Hokkaido University are essentially similar to those described in ref. ^49^. The composition of the Geological Survey of Japan reference material JB-3 is measured during the course of this study. Measured and reference values are listed in Table A1 of ref. ^49^.

### Electron microprobe analyses

Mineral compositions are determined using a JEOL JXA-8800 electron microprobe at Hokkaido University. For mafic minerals, an accelerating voltage of 15 kV, a beam current of 20 nA, and a counting time of 20 s are adopted. An accelerating voltage of 15 kV, a beam current of 10 nA, a counting time of 10 s, and a beam diameter of 10 μm are the operating conditions used for the plagioclase. Both oxide and natural mineral standards are used, and data are obtained using the ZAF correction method.

### Water contents of the dacitic melt

The water content of the dacitic magma is estimated using the plagioclase-melt hygrometer given in ref. ^50^. Plagioclase phenocrysts in the dacitic lavas are considered to have crystallised *in situ*, as it is observed that the lavas have no Eu anomaly (Fig. 2d), and because the ^87^Sr/^86^Sr ratios of the plagioclase phenocrysts are identical to those of the lavas (Fig. 4a). Therefore, it is assumed that the plagioclase phenocrysts with the highest An contents of 63.8 are in equilibrium with the melt that had a whole-rock composition of OS-2. The pressure condition is estimated using the compositions of the clinopyroxene and orthopyroxene phenocrysts, suggested to have grown simultaneously. By applying the two-pyroxene geobarometer given in ref. ^50^, we obtained a crystallisation pressure of 0.46 ± 0.4 GPa. The water content of the melt and the crystallisation temperature of plagioclase phenocrysts are then estimated by simultaneously solving Equations (25b) and (26) given in ref. ^50^ at a pressure of 0.46 GPa, to obtain a water content and a temperature of 5.3 ± 1.1 wt.% and 980 °C, respectively. The water contents of the dacites are also estimated by the plagioclase-melt hygrometer given in ref. ^51^, yielding 4.3 wt.%. This result is within the ranges of the estimates of 5.3 ± 1.1 wt.%, obtained by the hygrometer given in ref. ^50^.

## Supplementary information

Supplementary information.

Supplementary information2.
