# Supplementary material for: A new concept for the genesis of felsic magma: the separation of slab-derived supercritical liquid
Source: Sci Rep. 2020 May 26;10:8698. doi: 10.1038/s41598-020-65641-6 (PMC7251093; doi:10.1038/s41598-020-65641-6)
Supplement: Supplementary file 1 — Supplementary information. [file 41598_2020_65641_MOESM1_ESM.pdf]

Supplementary Information

Article in *Scientific Reports*

A new concept for the genesis of felsic magma: the separation of slab-derived supercritical liquid

Hajime Taniuchi[1]*, Takeshi Kuritani[2, 3], Tetsuya Yokoyama[3, 4], Eizo Nakamura[3], and Mitsuhiro Nakagawa[2]

[1] Department of Natural History Science, Graduate School of Science, Hokkaido University, Sapporo, Hokkaido, 060-0810, Japan

[2]Department of Earth and Planetary Science, Faculty of Science, Hokkaido University, Sapporo, Hokkaido, 060-0810, Japan

3 The Pheasant Memorial Laboratory, Institute for Planetary Materials, Okayama University, Misasa, Tottori, 682-0193, Japan

4 Department of Earth and Planetary Sciences, Tokyo Institute of Technology, Ookayama, Meguro, Tokyo, 152-8551, Japan

* Corresponding author

Telephone: +81-(0)11-706-4655

FAX: +81-(0)11-706-4658

e-mail: hajimetani@frontier.hokudai.ac.jp

Supplementary Figures

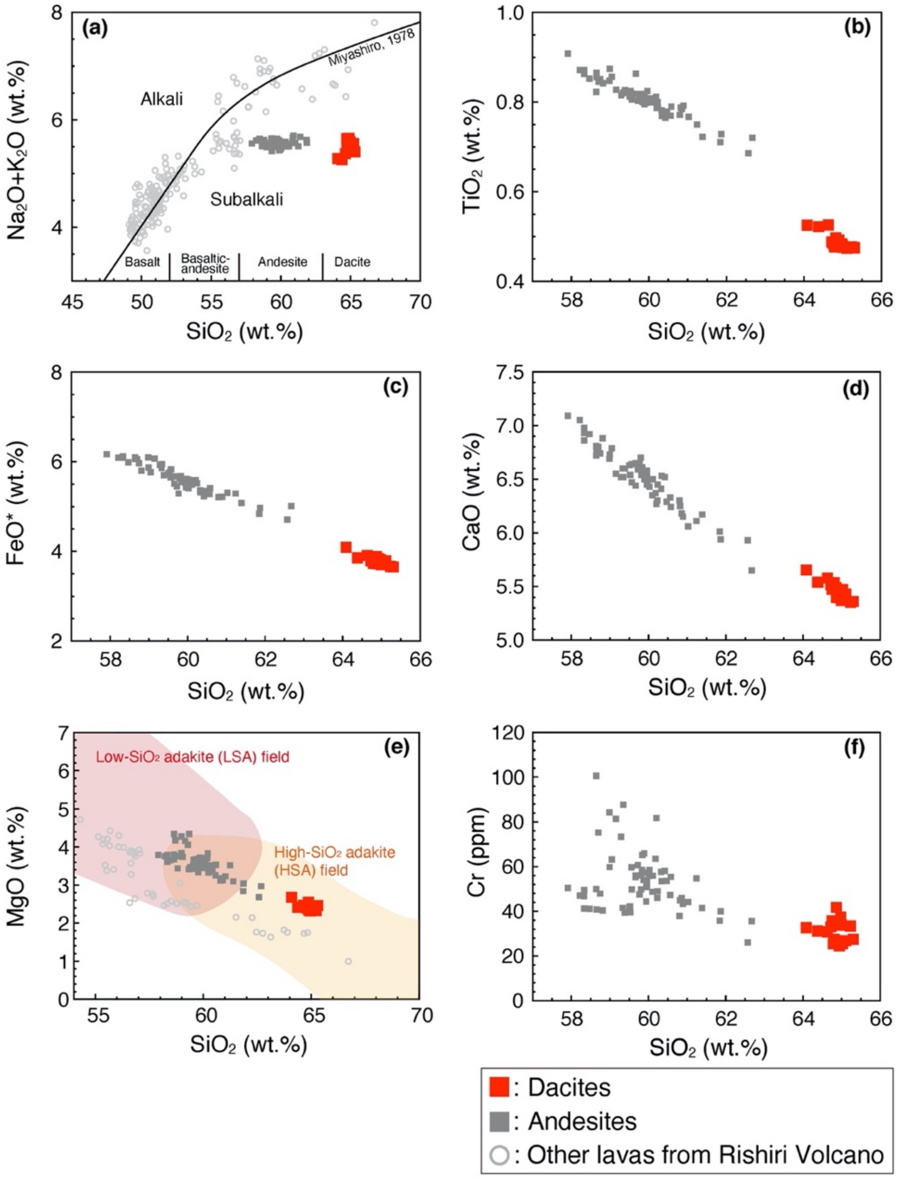

Figure S1. The whole-rock compositions of the dacite and the andesite lavas from Rishiri Volcano, shown in (a) Na_2O+K_2O–SiO_2 diagram, (b) TiO_2–SiO_2 diagram, (c) FeO*–SiO_2 diagram, (d) CaO–SiO_2 diagram, (e) MgO–SiO_2 diagram, and (f) Cr–SiO_2 diagram. In (a), discrimination line between alkali and sub-alkali series is taken from ref. [1]. In (e), the compositional fields of the Low-SiO_2 adakite (LSA) and High-SiO_2 adakite (HSA) are taken from ref. [2]. In (a)–(f), the whole-rock composition of the andesites and other lavas from Rishiri of ref. [3] and ref. [4], respectively, are also shown.

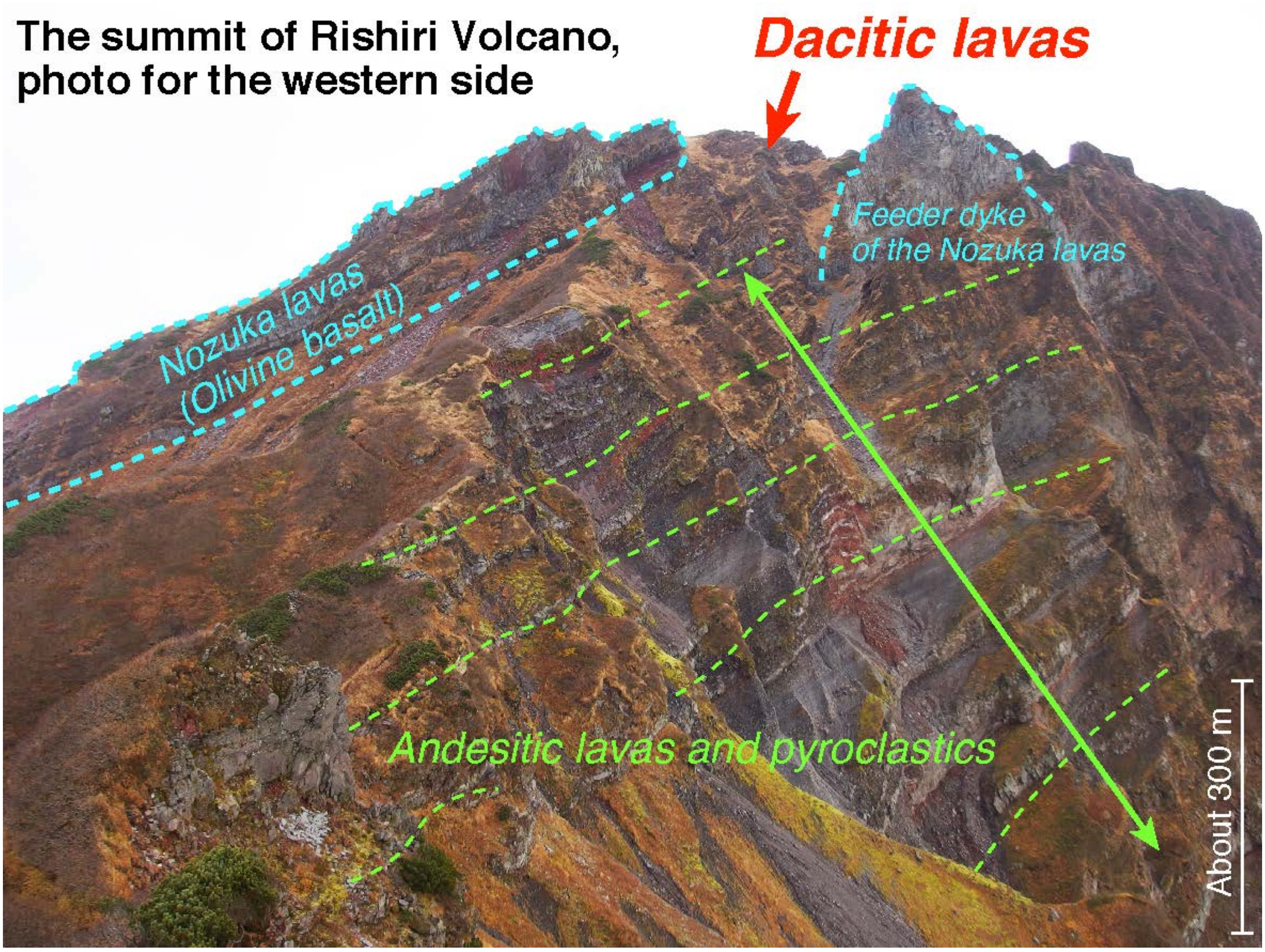

Figure S2. Photograph showing the calc-alkaline andesitic lavas and pyroclastics conformably overlain by the dacitic lavas. Nozuka lavas and a feeder dyke, erupted after the dacitic lavas (28,230 ± 1,020 y. BP; ref. [5]), are also shown.

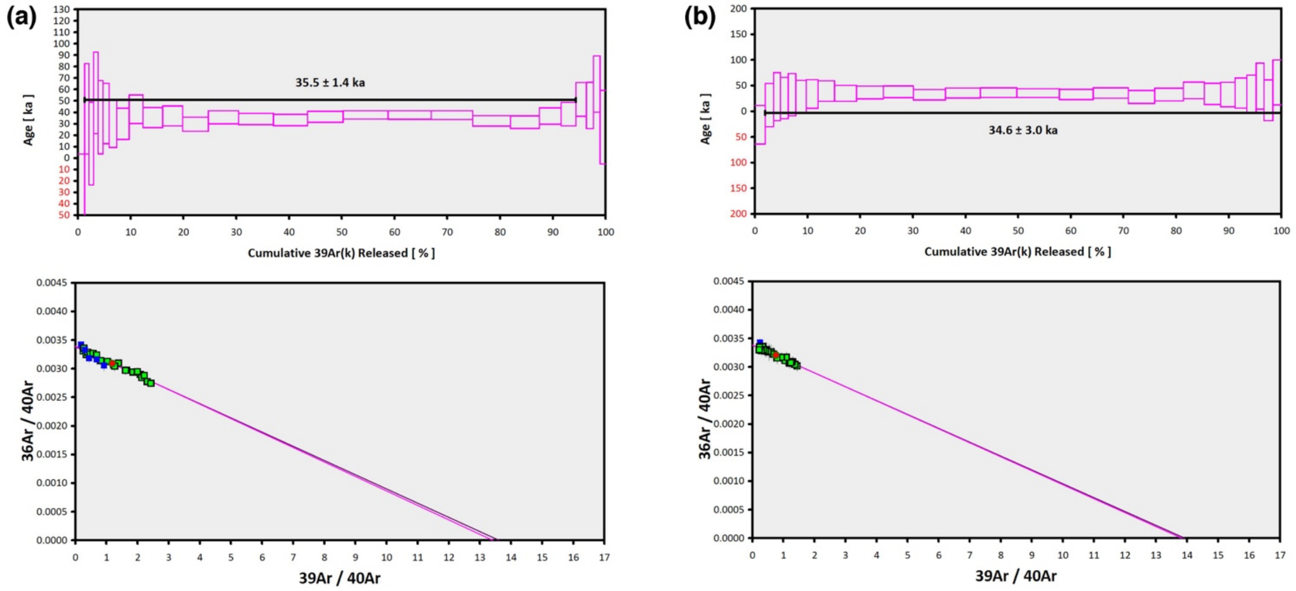

Figure S3. Representative age spectra (plateaus) and isochron plots derived from the $^{40}Ar/^{39}Ar$ incremental heating experiments on (a) the dacitic lavas and (b) the andesitic lavas from Rishiri Volcano. Plateau age (±1σ) is calculated from the weighted mean of the individual steps whose width is the proportion of total sample ^{39}Ar released and whose height is 2σ error.

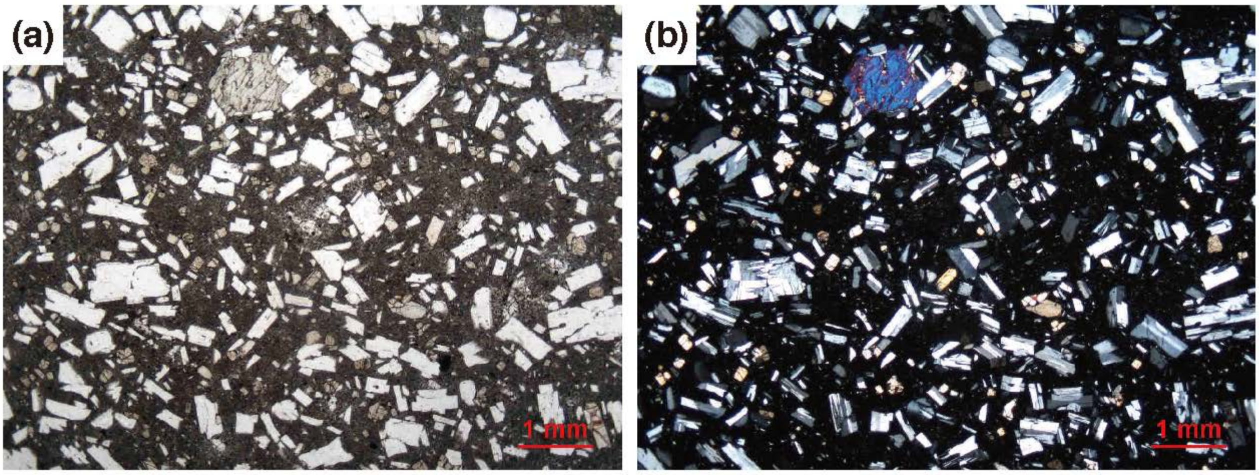

Figure S4. Photomicrographs of representative samples of (a and b) the dacite lava (parallel-and cross-polarized light, respectively).

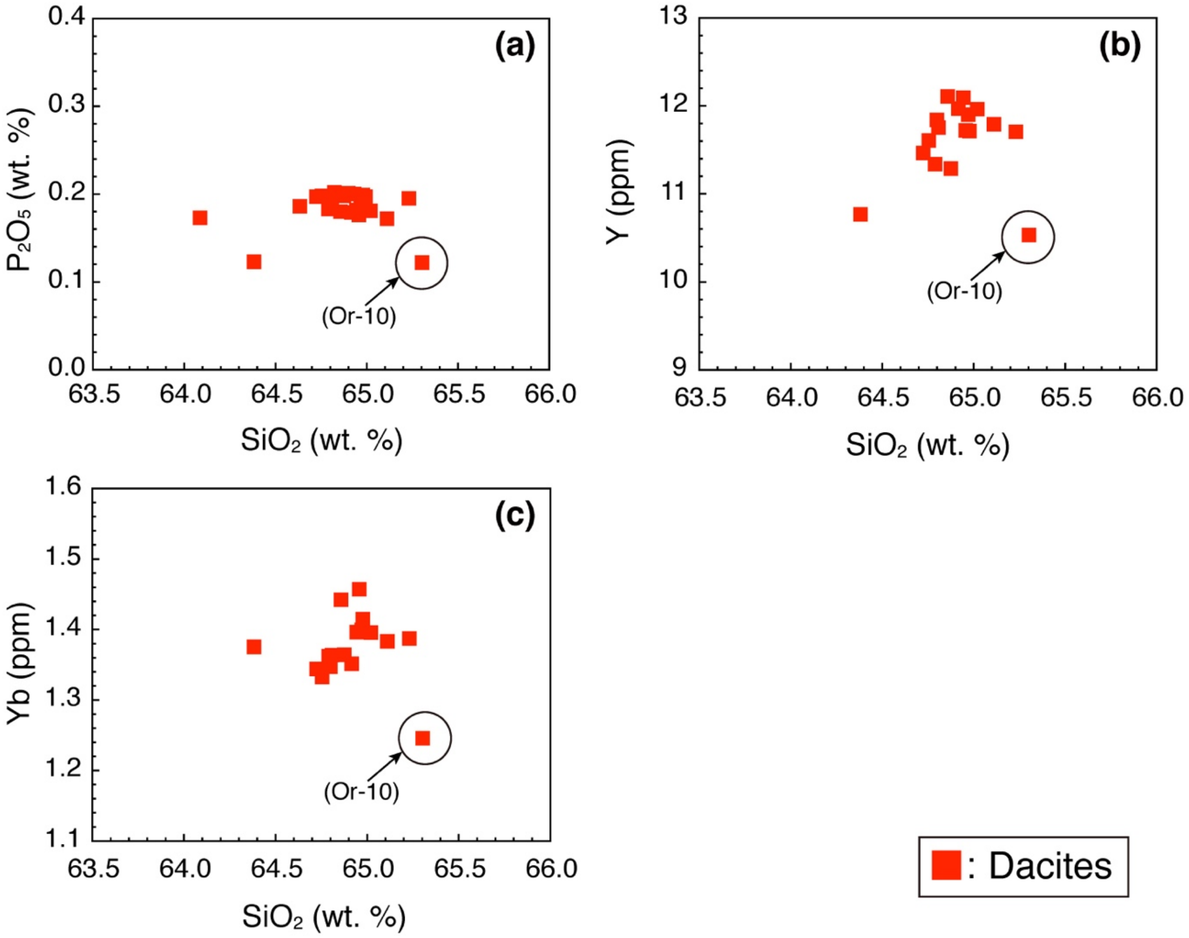

Figure S5. The whole-rock compositions of the dacite lavas from Rishiri Volcano, shown in (a) P_2O_5–SiO_2 diagram, (b) Y–SiO_2 diagram, and (c) Yb–SiO_2 diagram. The outlying dacite sample (Or-10) with a positive Eu anomaly in Fig. 2d has exceptionally low P_2O_5, Y, and Yb contents compared with those of other dacite samples. This magma may have undergone additional magmatic processes, such as plagioclase accumulation and apatite fractionation.

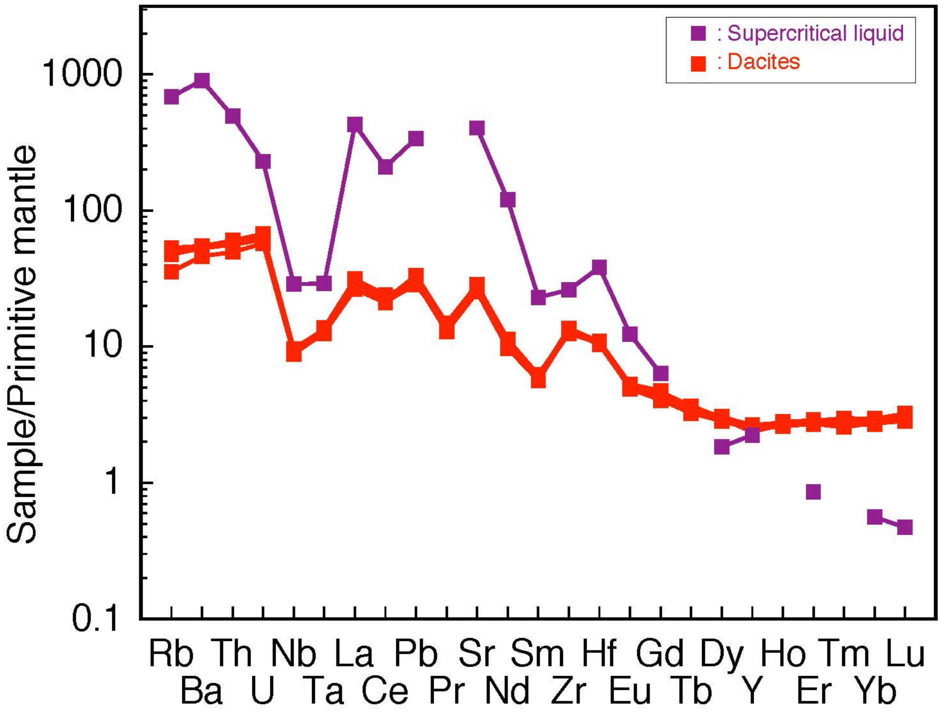

Figure S6. Primitive mantle-normalized trace element concentrations of possible slab-derived supercritical liquid and hydrous melt, along with those of the dacite. The trace element concentrations of the primitive mantle are taken from ref. [6].

Supplementary Methods

Estimation of chemical composition of slab-derived hydrous melt and supercritical liquid. The trace element compositions of slab-derived supercritical liquid are estimated using the compositions of the slab and the bulk partition coefficients of the elements between the slab and the supercritical liquid given in ref. [7]. The trace element compositions of the slab, at which the slab-derived materials (hydrous melt or supercritical liquid) are released, are obtained using the constraint of Pb isotopic ratios. The relative contribution of the sediment (SED) and the altered oceanic crust (AOC) components to the slab-derived materials can be estimated using the Pb concentration and the Pb isotopic ratio of the depleted MORB mantle (DMM) of ref. [8], those of SED and AOC component of ref. [9], and the Pb isotopic ratios of the dacitic lavas, from which a mixing proportion of AOC and SED of 5:95 was obtained. The trace element contents of the slab are then calculated using the mixing ratio and the trace element contents of the AOC [10] and SED [11]. The trace element contents of the slab-derived materials are finally estimated using the compositions of the slab and the partition coefficients of the elements ref. [7], and are displayed in a primitive mantle-normalized trace element concentration diagram (Fig. S6).

References

1. Miyashiro, A. Nature of alkalic volcanic rock series. *Contrib. Mineral. Petrol.* **66,** 91–104 (1978).
2. Martin, H. An overview of adakite, tonalite–trondhjemite–granodiorite (TTG), and sanukitoid: relationships and some implications for crustal evolution. *Lithos* **79,** 1–24 (2005).
3. Taniuchi, H., Kuritani, T. & Nakagawa, M. Generation of calc-alkaline andesite magma through crustal melting induced by emplacement of mantle-derived water-rich primary magma: Evidence from Rishiri Volcano, southern Kuril Arc. *Lithos* ***354–355***, 105362 (2020).
4. Ishizuka, Y. Mantle diaper model for polygenetic volcanoes: Geological and petrological study of Rishiri Volcano, northern Hokkaido, Japan. *PhD thesis, Grad. Sch. of Sci., Hokkaido Univ. Sapporo* (2000).
5. Miura, H. & Takaoka, S. Significance of the radiocarbon age and the identification of the fossil wood under lava flows erupted from Rishiri Volcano, Hokkaido, Japan. *Quat. Res.* **32,** 107–114 (1993).
6. Sun, S.-S., & McDonough, M. F. Chemical and isotopic systematics of oceanic basalts: Implications for mantle composition and processes. in *Magmatism in the Ocean Basins*, edited by A. D. Saunders and M. J. Norry, Geol. Soc. London Spec. Publ. **42,** 313–345 (1989).
7. Kessel, R., Schmidt, M. W., Ulmer, P. & Pettke, T. Trace element signature of subduction-zone fluids, melts and supercritical liquids at 120-180 km depth. *Nature* **437,** 724–727 (2005).

8. Cousens, B. L., & Allan, J. F. A Pb, Sr, and Nd isotopic study of basaltic rocks from the Sea of Japan, LEGS 127/128, in *Proceedings of Ocean Drilling Program Scientific Results* **127-128,** edited by K. Tamaki, et al., pp. 805–817, Ocean Drill. Program, College Station, Tex (1992).

9. Hauff, F., Hoernle, K. & Schmidt, A. Sr–Nd–Pb composition of Mesozoic Pacific oceanic crust (Site 1149 and 801, ODP Leg 185): Implications for alteration of oceanic crust and the input into the Izu–Bonin–Mariana subduction system. *Geochem. Geophys. Geosyst.* **4,** 8913, doi:10.1029/2002GC000421 (2003).

10. Pearce, J. A. & Parkinson, I. J. Trace element models for mantle melting: application to volcanic arc petrogenesis. In: Prichard, H. M., Alabaster, T., Harris, N. B. W. & Neary, C. R. (eds) *Magmatic Processes and Plate Tectonics. Geological Society, London, Special Publications,* **76,** 373–403 (1993).

11. Taylor, S. R. & McLennan, S. M. *The Continental Crust: its Composition and Evolution.* Oxford: Blackwell Scientific (1985).
